# A new species of *Nigorella* Wesołowska & Tomasiewicz, 2008 (Araneae, Salticidae, Salticinae, Plexippini) from Guangxi, China

**DOI:** 10.3897/BDJ.13.e158414

**Published:** 2025-06-13

**Authors:** Siqi Liu, Hong Liu, Feng Zhang

**Affiliations:** 1 Key Laboratory of Zoological Systematics and Application, College of Life Sciences, Hebei University, Baoding, Hebei 071002, China Key Laboratory of Zoological Systematics and Application, College of Life Sciences, Hebei University Baoding, Hebei 071002 China; 2 Hebei Basic Science Center for Biotic Interaction, Baoding, Hebei 071002, China Hebei Basic Science Center for Biotic Interaction Baoding, Hebei 071002 China; 3 Administration of Huaping National Nature Reserve, Guilin, Guangxi 541199, China Administration of Huaping National Nature Reserve Guilin, Guangxi 541199 China

**Keywords:** *Evarcha*, jumping spider, morphology, Plexippina, taxonomy

## Abstract

**Background:**

The genus *Nigorella* Wesołowska & Tomasiewicz, 2008 currently contains nine species, four of which are found in Africa and five in Asia (four in China and one in Thailand). The four Chinese species and their distribution in China are as follows: *Nigorellahirticeps* (Song & Chai, 1992), in Hunan and Hubei; *N.mengla* Lin & Li, 2020, in Yunnan; *N.orientalis* (Song & Chai, 1992), in Hubei, Sichuan and Guizhou; and *N.sichuanensis* (Peng, Xie & Kim, 1993), in Sichuan.

**New information:**

A new species of *Nigorella* is described, based on both sexes (two male and four female specimens) from the Huaping National Nature Reserve, Guangxi, China: *Nigorellahuaping* Liu & Zhang, **sp. nov.**

## Introduction

The genus *Nigorella* Wesołowska & Tomasiewicz, 2008 was originally established to include four African species, with the type species being *Nigorellaaethiopica* Wesołowska & Tomasiewicz, 2008 from Ethiopia (Wesołowska and Tomasiewicz 2008). The genus was placed in the subtribe Plexippina of the tribe Plexippini (Maddison 2015). Subsequently, four Asian species of *Evarcha* Simon, 1902, including three from China: *E.hirticeps* (Song & Chai, 1992), *E.hunanensis* Peng, Xie & Kim, 1993 and *E.sichuanensis* Peng, Xie & Kim, 1993 and one from Thailand: *E.petrae* Prószyński, 1992, were transferred to *Nigorella* due to their resemblance in genital characters, even though this taxonomic treatment was noted as tentative (Song and Chai 1992, Prószyński 2018). Subsequent studies (Lin and Li 2020, Lin et al. 2023) confirmed that *N.hunanensis* is a junior synonym of *N.hirticeps*, transferred *Evarchaorientalis* (Song & Chai, 1992) to *Nigorella* and described *Nigorellamengla* Lin & Li, 2020 from Xishuangbanna, Yunnan. Despite these developments, there has been little attention given to the taxonomy of *Nigorella* species in China.

The Huaping National Nature Reserve (NNR Huaping, 25°31'–25°36' N, 109°48'–109°58'E) of northern Guangxi, is amongst the earliest established nature reserves in China, having been founded for over five decades. Additional baseline information and environmental profiles of this Reserve was provided in the survey report by Kadoorie Farm and Botanic Garden (Kadoorie Farm and Botanic Garden 2002). Over the past two decades, systematic surveys have documented rich fauna of this region, with ongoing discoveries of novel species in recent years （Kadoorie Farm and Botanic Garden 2002, Hormiga and Tu 2008, Wang et al. 2020, Li et al. 2021, Luo et al. 2023, Liao et al. 2024, Li et al. 2024, Peng and Li 2024, World Spider Catalog 2025). Despite these advancements, reports on the spiders of this area remain rather limited.

During recent surveys of jumping spiders in the NNR Huaping, we discovered an undescribed species of Plexippina. This species was assigned to the genus *Nigorella* due to its striking genital similarities with *Nigorellahirticeps*. In this study, we formally describe this newly-discovered species as *Nigorellahuaping* Liu & Zhang, **sp. nov.** This description is part of our ongoing efforts to document and understand the diversity of jumping spiders in the Huaping Region.

## Materials and methods

All specimens were preserved in 75% ethanol, examined and measured under a Leica M205A stereo-microscope. All measurements are given in millimeters (mm). Photographs of specimens were taken using an Olympus BX51 microscope, equipped with a Kuy Nice CCD camera or a Leica M205A stereomicroscope, equipped with a Leica DFC550 CCD camera and were imported into Helicon Focus v. 8 or LAS V4.3 software for stacking. Final figures were retouched in Adobe Photoshop 2023. All type specimens examined are deposited in the Museum of Hebei University (MHBU; Baoding, China).

Abbreviations: **AC**, apical cymbial groove; **ALE**, anterior lateral eye; **AME**, anterior median eye; **At**, atrium; **ar**, anterior rim of atrium; **CTA**, compound terminal apophysis of embolus; **CO**, copulatory opening; **Cy**, cymbium; **E**, embolus; **FD**, fertilization duct; **P**, pocket; **pd**, spine on pro-dorsal side of leg segment; **PLE**, posterior lateral eye; **PME**, posterior median eye; **pv**, spine on pro-ventral side of leg segment; **rs**, ridge of septum; **rv**, spine on retro-ventral side of leg segment; **RTA**, retrolateral tibial apophysis; **S**, spermatheca; **SM**, spermophor; **St**, septum; **TL**, tegular lobe; **Ti**, tibia; **v**, spine on ventral side of leg segment.

## Taxon treatments

### Nigorella huaping

Liu & Zhang sp. nov.

D9DDFDB3-E85B-51E2-9F97-E06332623B78

5ECA33F9-B657-41DE-91A9-5E228FEDEC5B

#### Materials

- **Type status:** Holotype. **Occurrence:** recordNumber: GX-16-15; recordedBy: Chi Jin, Jingchao He & Beibei Zhou; individualCount: 1; sex: male; lifeStage: adult; occurrenceID: 56AB968D-1EE0-5342-9652-6552D9902813; **Taxon:** order: Araneae; family: Salticidae; genus: Nigorella; **Location:** country: China; stateProvince: Guangxi; locality: Longsheng Autonomous Country, Sanmen Township, Huaping Reserve; verbatimElevation: 894 m; verbatimLatitude: 25°37'09.80''N; verbatimLongitude: 109°55'54.50''E; **Event:** year: 2016; month: 6; day: 22; **Record Level:** institutionCode: MHBU-ARA-00020080
- **Type status:** Paratype. **Occurrence:** recordNumber: GX-16-15; recordedBy: Chi Jin, Jingchao He & Beibei Zhou; individualCount: 1; sex: female; lifeStage: adult; occurrenceID: C2E42670-2989-5DF1-9FE8-352A1265CFCB; **Taxon:** order: Araneae; family: Salticidae; genus: Nigorella; **Location:** country: China; stateProvince: Guangxi; locality: Longsheng Autonomous Country, Sanmen Township, Huaping Reserve; verbatimElevation: 894 m; verbatimLatitude: 25°37'09.80''N; verbatimLongitude: 109°55'54.50''E; **Event:** year: 2016; month: 6; day: 22; **Record Level:** institutionCode: MHBU-ARA-00020617
- **Type status:** Paratype. **Occurrence:** recordNumber: GX-16-14; recordedBy: Chi Jin, Jingchao He & Beibei Zhou; individualCount: 1; sex: female; lifeStage: adult; occurrenceID: 5A200F30-E185-5082-A5A8-43D46145A09D; **Taxon:** order: Araneae; family: Salticidae; genus: Nigorella; **Location:** country: China; stateProvince: Guangxi; locality: Longsheng Autonomous Country, Sanmen Township, Huaping Reserve; verbatimElevation: 785 m; verbatimLatitude: 25°37'71''N; verbatimLongitude: 109°55'09.50''E; **Event:** year: 2016; month: 6; day: 20; **Record Level:** institutionCode: MHBU-ARA-00020651
- **Type status:** Paratype. **Occurrence:** recordNumber: GX-16-14; recordedBy: Chi Jin, Jingchao He & Beibei Zhou; individualCount: 1; sex: female; lifeStage: adult; occurrenceID: FBCD177E-CEE8-5FAA-BBBF-65BC7B483C8A; **Taxon:** order: Araneae; family: Salticidae; genus: Nigorella; **Location:** country: China; stateProvince: Guangxi; locality: Longsheng Autonomous Country, Sanmen Township, Huaping Reserve; verbatimElevation: 785 m; verbatimLatitude: 25°37'71''N; verbatimLongitude: 109°55'09.50''E; **Event:** year: 2016; month: 6; day: 20; **Record Level:** institutionCode: MHBU-ARA-00020852
- **Type status:** Paratype. **Occurrence:** recordNumber: GX-16-15; recordedBy: Chi Jin, Jingchao He & Beibei Zhou; individualCount: 1; sex: male; lifeStage: adult; occurrenceID: 3E7C3AA6-C940-5761-BB38-E15FCB389545; **Taxon:** order: Araneae; family: Salticidae; genus: Nigorella; **Location:** country: China; stateProvince: Guangxi; locality: Longsheng Autonomous Country, Sanmen Township, Huaping Reserve; verbatimElevation: 894 m; verbatimLatitude: 25°37'09.80''N; verbatimLongitude: 109°55'54.50''E; **Event:** year: 2016; month: 6; day: 22; **Record Level:** institutionCode: MHBU-ARA-00028052
- **Type status:** Paratype. **Occurrence:** recordNumber: HBUARA#2023-55; recordedBy: Zhiyong Yang; individualCount: 1; sex: female; lifeStage: adult; occurrenceID: BFC7750F-78E7-56D4-9A5D-B2CC97B990B1; **Taxon:** order: Araneae; family: Salticidae; genus: Nigorella; **Location:** country: China; stateProvince: Guangxi; locality: Longsheng Autonomous Country, Sanmen Township, Huaping Reserve; verbatimElevation: 464.95 m; verbatimLatitude: 25°38'11.47''N; verbatimLongitude: 109°54′16.19''E; **Event:** year: 2023; month: 4; day: 29; **Record Level:** institutionCode: MHBU-ARA-00028053

#### Description

**Male** (Holotype). **Measurements.** Total length 7.34. Carapace 3.41 long, 2.57 wide. Abdomen 3.95 long, 2.16 wide. Eye measurements: AME 0.62, ALE 0.29, PME 0.06, PLE 0.24. Leg measurements: I 6.64 (1.96, 0.91, 1.73, 1.27, 0.77), II 6.27 (2.04, 0.93, 1.62, 0.98, 0.70), III 6.79 (2.10, 1.13, 1.34, 1.15, 1.07), IV 6.60 (2.11, 1.40, 1.41, 0.81, 0.87); leg formula 3412. **Carapace.** Dark, area postrior to fovea slightly lighter than other area, setae sparse (Fig. 2A). **Abdomen.** Elongated oval, lead-grey in ethanol; dorsal side covered by pale scales in anterior portion and lateral sides; with dorsal scutum (Fig. 2A). **Legs.** Dark brown, bearing long and thin spines; metatarsus light yellow. **Leg spination.** Femur Ⅰ: pd 1 (1/3 down from distal); Tibia I: pv 2; metatarsus I: pv 2, v 1 (on distal); femur Ⅱ: pd 1 (1/2 down from distal); tibia Ⅱ: v 2 (on distal); metatarsus Ⅱ: pv 1, rv 2; femur Ⅲ: pd 1 (1/3 down from distal); tibia Ⅲ: pv 1, rv1, v 1 (on distal); metatarsus Ⅲ: pv 2, rv 1; femur Ⅳ: pd 1 (1/3 down from distal), rv 1; tibia Ⅳ: pv 2, rv, 2; v 1; metatarsus Ⅳ: pv 3, rv 2; other segments leg parts spineless. **Palp.** With dense dark and long setae on dorsal of patella, tibia and pro-dorsal side of proximal of cymbium, cymbium flattened. Embolus curved and stout, compound terminal apophysis (CTA) flattened and closed to distal part of embolus. Retrolateral tibial apophysis (RTA) base broad, bifurcated distally. Tegular lobe (TL) well developed, pointing downwards, with stout ending (Fig. 2F, G, Fig. 3C and D).

**Female** (paratype, MHBU-ARA-00020617). **Measurements.** Total length 7.53. Carapace 3.25 long, 2.32 wide. Abdomen 4.00 long, 2.35 wide. Eye measurements: AME 0.64, ALE 0.26, PME 0.06, PLE 0.22. Leg measurements: I 4.93 (1.54, 0.88, 1.18, 0.74, 0.59), II 4.27 (1.45, 0.90, 0.97, 0.57, 0.38), III 5.70 (1.86, 1.01, 0.95, 1.09, 0.79), IV 5.02 (1.47, 0.70, 1.17, 1.10, 0.58); leg formula 3412. Overall pattern like male, abdomen lacking dorsal scutum, dorsal side has white areas on both sides (Fig. 2B). **Chelicerae.** Black brown, with one retromarginal tooth and two promarginal teeth (Fig. 2E); endites and labium dark. **Leg spination.** Femur I: pd 1 (1/3 down from distal), rv 1; tibia I: rv 3; metatarsus I: rv 1; Femur II: pd 1 (1/3 down from distal), rv 2; tibia II: rv 2; femur III: pd 1 (1/3 down from distal); tibia II: pv 3, rv 1; metatarsus III: pv 2, rv 2; Femur Ⅳ: pd 1 (1/4 down from distal); tibia Ⅳ: pv 3, rv 2; other segments of legs spineless. **Epigyne** (Fig. 3A–B and E–F) 1.5 times longer than wide, with pair of hoods near epigastral furrow; atria oval, with distinct anterior rim (ar) and ridge of the septum (rs); copulatory openings locating in anterior portion of atria; spermathecae thick V-shaped, with slightly twisted inner tubes; fertilization ducts well-developed and close to proximal position of spermathecae.

#### Diagnosis

The new species is similar to *Nigorellahirticeps* (Song & Chai, 1992) in the genital morphology, but it can be distinguished from the latter by the following: (1) the dorsal surface of abdomen in males with scutum (Fig. 2A, vs. in *N.hirticeps*, such scutum is absent; see Lin et al. (2023): fig. 12A); (2) the embolus is upturned distally (Fig. 2F and 3C; vs. in *N.hirticeps*, the embolus extends gently towards the distal end); (3) the compound terminal apophysis of the embolus (CTA) turns smoothly to the retrolateral side, forming no ledge-like extension visible from the ventral view (Fig. 2F and 3C; in *N.hirticeps*, the CTA forms a small, ventrally visible ledge-like extension; see Lin et al. (2023): fig. 11A); (4) the female atria have obvious anterior rims (ar; Fig. 2 A and E; vs. in *N.hirticeps*, such rims are absent; see Zha and Zhang (2014): fig. 9); (5) the spermathecae have lateral extensions distally (Fig. 3 B and F; vs. such extensions are absent in *N.hirticeps*; see Zha and Zhang (2014): fig. 10).

#### Etymology

The specific epithet is derived from the type locality and is a noun in apposition.

#### Distribution

**Figure 1.**
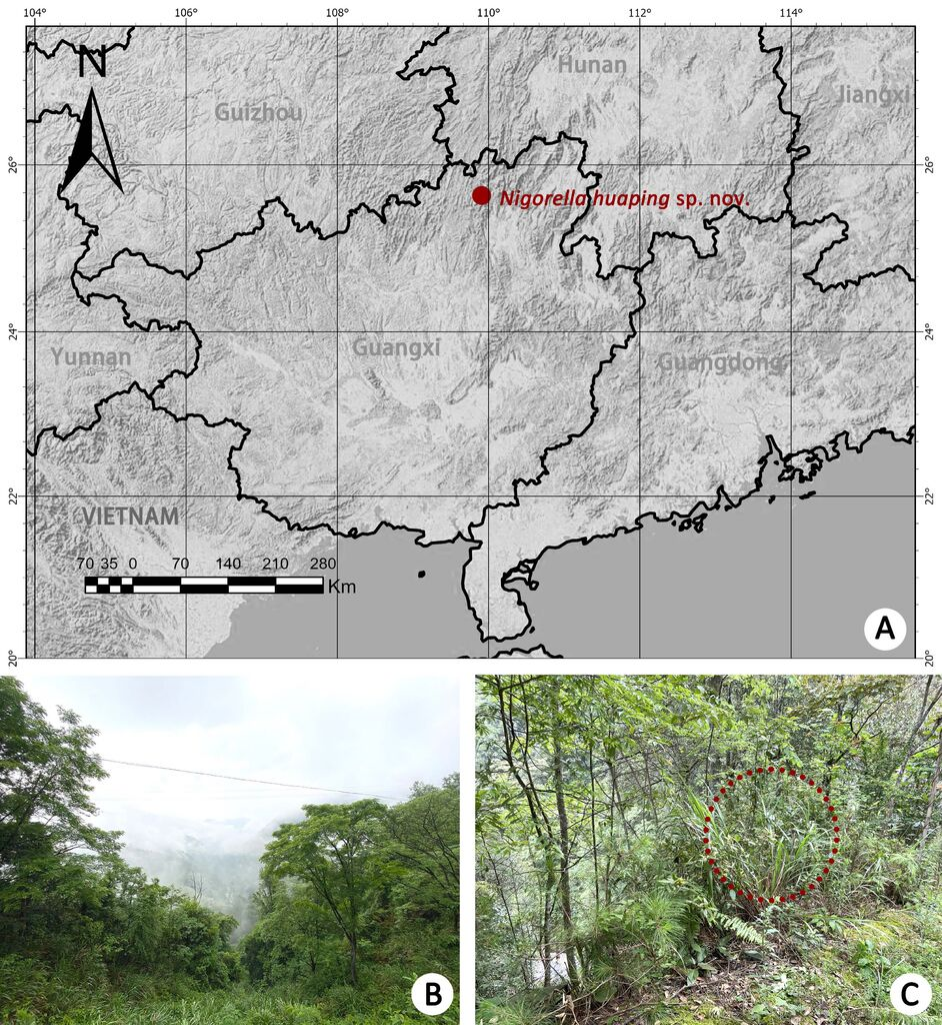
Distribution (**A**) and habitats (**B, C**) of *Nigorellahuaping* **sp. nov.** from Guangxi, China. The red circle in **C** indicates where a male was collected.

**Figure 2.**
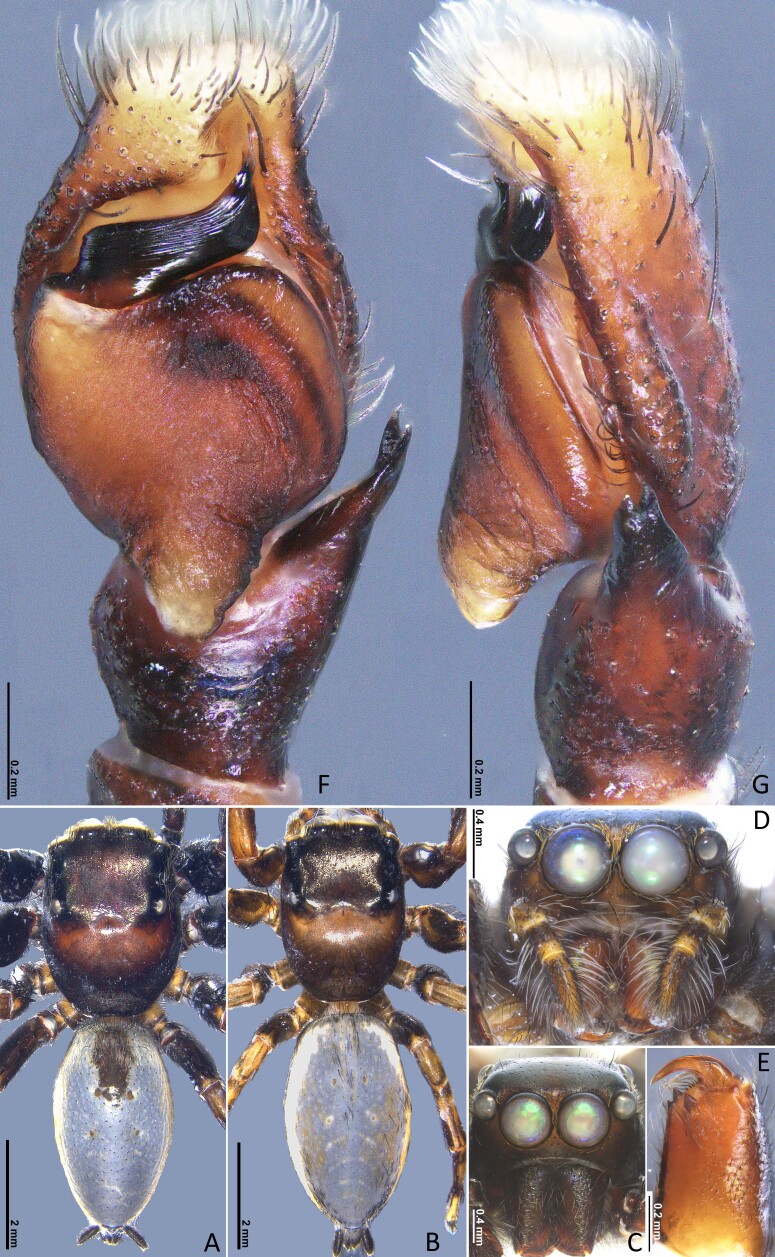
*Nigorellahuaping* **sp. nov.**, holotype male (**A, C, F, G**) and paratype female (MHBU-ARA-00020617) (**B, D, E**). **A, B** Habitus; **C, D** Front view of carapace and chelicerae; **E** Cheliceral teeth; **F, G** Left palp; in dorsal (**A, B**), front (**C, D**), ventral (**F**) and retrolateral (**E, G**) view.

**Figure 3.**
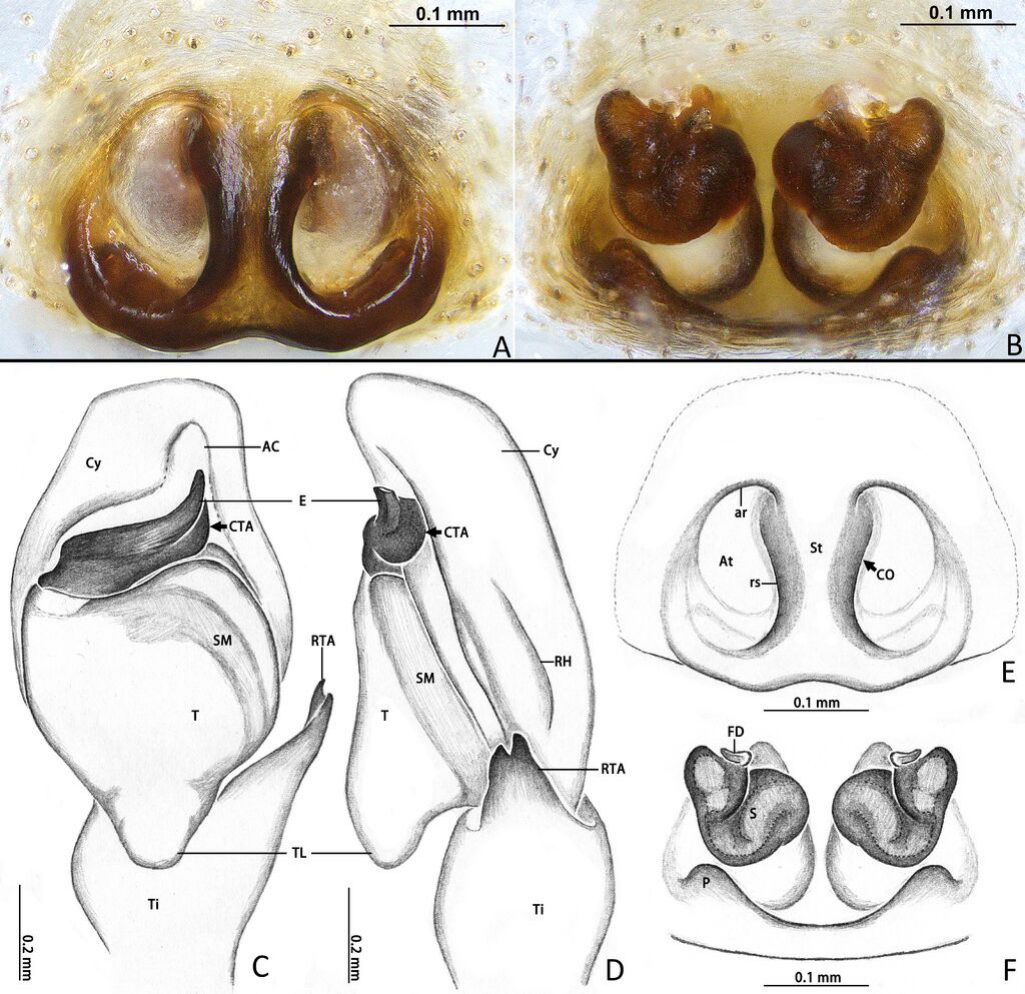
*Nigorellahuaping* **sp. nov.**, copulatory organ (**A, B, E, F**) of paratype female (MHBU-ARA-00020617) and palp of holotype male **(C, D**); in ventral (**A, C, E**), dorsal (**B, F**) and retrolateral (**D**) view. Abbreviations: AC, apical cymbial groove; At, atrium; ar, anterior rim of atrium; CTA, compound terminal apophysis of embolus; CO, copulatory opening; Cy, cymbium; E, embolus; FD, fertilization duct; P, pocket; RTA, retrolateraliz tibial apophysis; rs, ridge of septum; S, spermatheca; SM, spermophor; St, septum; TL, tegular lobe; Ti, tibia.

Type locality in Guangxi, China (Fig. 1).

## Supplementary Material

XML Treatment for Nigorella huaping
